# Macrophages as Key Drivers of Cancer Progression and Metastasis

**DOI:** 10.1155/2017/9624760

**Published:** 2017-01-22

**Authors:** Sebastian R. Nielsen, Michael C. Schmid

**Affiliations:** Department of Molecular and Clinical Cancer Medicine, University of Liverpool, Ashton Street, Liverpool L69 3GE, UK

## Abstract

Macrophages are one of the most abundant immune cells in the tumour microenvironment of solid tumours and their presence correlates with reduced survival in most cancers. Macrophages are present at all stages of tumour progression and stimulate angiogenesis, tumour cell invasion, and intravasation at the primary site. At the metastatic site, macrophages and monocytes prepare for the arrival of disseminated tumour cells and promote their extravasation and survival by inhibiting immune-mediated clearance or by directly engaging with tumour cells to activate prosurvival signalling pathways. In addition, macrophages promote the growth of disseminated tumour cells at the metastatic site by organising the formation of a supportive metastatic niche. The development of agents inhibiting the recruitment or the protumorigenic effector functions of macrophages in both the primary tumour and at the metastatic site is a promising strategy to improve cancer survival in the future.

## 1. Macrophage Origin in Healthy Tissues and the Tumour Microenvironment

Monocytes and macrophages are a subset of leukocytes that play distinct roles in tissue homeostasis and immunity. In general, monocytes are important during inflammation and pathogen challenge, whereas tissue-resident macrophages have important roles in development, homeostasis, and resolution of inflammation [1]. Some of the homeostatic functions of tissue-resident macrophages include regulation of angiogenesis and removal of apoptotic cells. Macrophages play a key role in the development of blood vessels, which has been mostly studied in the retina, specifically by promoting endothelial tip cell anastomosis and by limiting excessive vessel sprouting [2–4]. In addition, macrophages remove apoptotic cells during limb formation and ingest the extruded erythrocyte nuclei during erythropoiesis. In addition, macrophages maintain hematopoietic steady state by engulfment of neutrophils and eosinophils in the liver and spleen [5]. During inflammatory responses, macrophages play a dual role by initial secretion of inflammatory mediators, including tumour necrosis factor alpha (TNF*α*) and interleukin (IL) 1 beta (IL-1*β*) and nitric oxide, which activate antimicrobial defence mechanisms that contribute to the killing of invading organisms. Although these inflammatory macrophages are initially beneficial, they also trigger substantial tissue damage and must be quickly controlled, if not they become pathogenic and contribute to disease progression. To balance the tissue-damaging potential of the inflammatory macrophage response, macrophages undergo apoptosis or switch into an anti-inflammatory phenotype that reduces the proinflammatory response while facilitating wound healing [5]. As an example, in liver fibrosis, selective depletion of macrophages during the fibrosis-promoting insult resulted in reduced fibrosis, whereas macrophage depletion after cessation of the insult delayed the fibrotic resolution [6].

Tissue-resident macrophages can develop from three independent sources during embryonic development and adulthood: yolk sac-derived macrophages and fetal liver-derived monocytes (embryonic) or hematopoietic stem cells in the bone marrow (adult). Most tissue-resident macrophages in the adult organism are derived from embryonic precursors that seed the tissues before birth during two waves of haematopoiesis. The first wave comprises macrophages that develop from early erythromyeloid progenitors in the yolk sac at embryonic age (E) 8.5–9.0 in mice. The second wave includes fetal liver monocytes, generated in the fetal liver from E12.5 onward after late yolk sac-derived erythromyeloid progenitors migrate into the fetal liver [1]. During the two waves of haematopoiesis, yolk sac-derived macrophages and fetal liver monocytes migrate to populate the embryonic tissue. Tissue-resident macrophages are capable of maintaining their populations through proliferation, which means that in the adult steady state organism, monocytes do not contribute to the maintenance of most peripheral tissue macrophages. This includes microglia in the brain, Kupffer cells in the liver, and Langerhans cells in the epidermis [7–10]. The third source of macrophages comes from hematopoietic stem cells that colonise the bone marrow from E17.5 onward and produce monocytes that seed the blood continuously throughout adult life. Fate mapping has identified yolk sac-derived macrophages as the main precursor for brain microglia [11], whereas fetal liver-derived monocytes are the main precursor of liver Kupffer cells and lung alveolar macrophages [12, 13], while bone marrow-derived monocytes replenish intestinal and cardiac macrophages in the steady state adult organism [14, 15].

Monocytes in the circulation can be differentiated into two subsets based on cell surface expression of different markers. Inflammatory monocytes are characterised by Ly6C^high^ CX3CR1^mid^ CCR2^+^ CD62L^+^ CD43^low^ (Ly6C^high^) expression, whereas patrolling monocytes are characterised by Ly6C^low^ CX3CR1^high^ CCR2^−^ CD62L^−^ CD43^high^ (Ly6C^low^) expression. Inflammatory monocytes are rapidly recruited to sites of inflammation, including cancer, by chemokines such as macrophage-colony stimulating factor 1 (CSF-1), C-C motif chemokine ligand 2 (CCL2), and stromal cell-derived factor 1 alpha (SDF-1*α*), where they extravasate from the blood vessels and differentiate into monocyte-derived macrophages [16–18]. In contrast, patrolling monocytes reside in the blood vessel lumen where they patrol the endothelial surface on the luminal side of the vessel and coordinate its repair through recruitment of neutrophils [19, 20].

The tumour microenvironment is a complex assembly of genetically heterogeneous cancer cells and the different cell types that constitutes the local environment. These cells include endothelial cells, cancer-associated fibroblasts, and different populations of immune cells. Macrophages are one of the most abundant immune cells in the tumour microenvironment of solid tumours [16, 21]. There is a strong correlation between the density of macrophages and poor survival in and carcinomas of pancreas, breast, lung, cervix, the bladder, and Hodgkin's lymphoma [22–26]. In addition, expression of CSF-1, the major lineage regulator for macrophages, or its receptor CSF-1R correlates with poor survival in liver and breast and pancreatic cancer [27, 28], respectively. Furthermore, a macrophage transcriptional signature in patients with breast cancer is predictive of poor prognosis and reduced survival [29, 30]. Using flow cytometry and different genetic mouse models, it was recently demonstrated in breast cancer that tumour growth was associated with a decrease in mammary tissue macrophages and an increase in tumour-associated macrophages (TAMs). These TAMs were distinguished from mammary-resident macrophages based on the surface expression of CD11b^low^ MHCII^high^ F4/80^+^ CD64^+^ MerTK^+^ on TAMs. Importantly, this TAM population was recruited directly from CCR2+ inflammatory monocytes that proliferated and differentiated into TAMs in the tumour microenvironment [31]. Flow cytometric analysis of myeloid populations in tumours suggests that Ly6C+ inflammatory monocytes are recruited from the blood circulation and the splenic reservoir and differentiate into Ly6C^low^ TAMs. These TAMs are heterogenous populations that can be further divided into separate populations based on high and low expression of MHC class II [17, 18]. Although these reports suggest that most TAM subpopulations arise from the Ly6C+ population of circulating mouse monocytes, the contributions of tissue-resident embryo-derived macrophages to TAM populations remain less well understood and might likely differ depending on the tumour type and localization. However, examples of markers used to identify monocytes and macrophages in development and disease can be seen in Table 1.

## 2. Macrophage and TAM Phenotypes

Macrophages display a high degree of adaptability in response to changes in their immediate environment. It was initially proposed that macrophages could be polarized into two distinct phenotypes based on their response to interferon gamma (IFN*γ*) and lipopolysaccharide (LPS) (termed M1 macrophages) or IL-4 and IL-13 (termed M2 macrophages). The M1 phenotype is associated with production of proinflammatory cytokines, such as IL-12, IFN*γ* and TNF*α*, antigen presentation, generation of reactive oxygen species, and the ability to eliminate pathogens and cells. In contrast, the M2 phenotype is associated with the production of anti-inflammatory cytokines, such as IL-10, upregulation of scavenging receptors, and tissue remodeling [32–34]. However, depending on the activation signals, macrophages can acquire different phenotypes and functions in which the M1 and M2 phenotypes represent the extremes of this spectrum. Stimulation with other factors such as IL-10, immune complexes, transforming growth factor *β* (TGF*β*), and glucocorticoids can promote macrophage M2 polarization into specific M2 subtypes that are distinct from the classical M2 phenotype induced by IL-4 [34]. Indeed, transcriptomic profiling of human monocyte-derived macrophages exposed to a wide variety of stimuli confirms that transcriptomic changes in M1 (stimulated by IFN*γ*) and M2 (stimulated by IL-4) macrophages are found at either end of a bipolar axis, in which stimulation with other factors associated with M1 (LPS, TNF*α*) or M2 (IL-13) macrophages does not change. In contrast, addition of other factors such as IL-10, free fatty acids, prostaglandin, or high-density lipoprotein reveals separate clusters of transcriptomic changes in macrophage activation along the bipolar M1/M2 axis [35].

TAMs are typically associated with an M2-like polarization state caused by tumour-derived lactic acid or secretion of immunosuppressive cytokines such as IL-4, IL-10, and IL-13 from different cells in the tumour microenvironment or B cell-derived immunoglobulins [36–40]. Hypoxia, a common feature of the tumour microenvironment in most cancers, does not influence TAM polarization directly. Instead, several reports confirm that heterogenous TAM populations are found in distinct compartments within tumours based on the level of hypoxia in these areas. TAMs are recruited to hypoxic tumour areas by cancer cell-derived VEGF-A and semaphorin 3A through VEGFR1/neuropillin-1 signalling. TAMs are retained inside the hypoxic areas to promote tumour angiogenesis by downregulation of neuropillin-1 and semaphorin 3A-mediated PlexinA1/A4 signalling. Interfering with neuropillin-1 in TAMs restricts their presence to oxygenated areas where they promote antitumour immunity and inhibit angiogenesis [41]. Inflammatory monocytes give rise to both MHCII^low^ and MHCII^high^ TAMs, but TAMs inside hypoxic regions were predominantly MHCII^low^ and associated with increased expression of M2-markers. Interestingly, hypoxia does not promote M2 polarization since there was no difference in MHCII^low^ and MHCII^high^ TAMs or their expression of M2 markers in well-oxygenated tumours. Instead, hypoxia primarily regulates the expression of genes that promotes angiogenesis. Thus, hypoxia primarily regulates a subset of M2-related genes that affects the tumour angiogenic phenotype of TAMs [42].

## 3. Tumour-Promoting Functions of TAMs

Macrophages display several protumorigenic functions that have important roles in cancer development and progression such as the ability to provide cytokines and induce tumour angiogenesis [43]. TAMs are a source of tumour-promoting IL-6 in several murine tumour models. Tumour-associated myeloid cell production of IL-6 promotes colon tumour cell proliferation and protection from apoptosis through activation of STAT3 [44, 45]. A similar effect is seen in pancreatic cancer, where myeloid-derived IL-6 promotes tumour progression from epithelial precursor lesions through STAT3 [46]. In a genetic model of colorectal cancer, tumour development is initiated through loss of the adenomatous polyposis coli tumour suppressor gene that leads to activation of *β*-catenin and results in disruption of the epithelial barrier. This allows microbial products to penetrate and induce the production of IL-23 from macrophages. IL-23 drives a Th17 response in CD4+ T cells through IL-6 and IL-17, which ultimately results in colorectal cancer progression [47].

Blood vessels in healthy tissues reside in a quiescent state where angiogenesis is only transiently activated in response to certain stimulus. In contrast, during tumour progression, an “angiogenic switch” is almost always activated and remains on, causing normally quiescent vasculature to continually sprout new vessels. However, compared to a normal vascular network, the blood vessels in tumours are characterised by convoluted and excessive vessel branching, distorted and enlarged vessels, erratic blood flow, microhemorrhaging, and leakiness [21]. Macrophages are important for this angiogenic switch in tumours particularly through production of vascular-endothelial growth factor A (VEGF-A) and placental growth factor (PlGF). In particular, the blood vessels in tumours lacking myeloid cell-derived VEGF-A were less tortuous, with increased pericyte coverage and decreased vessel length. These are all characteristics that indicate a normalization of the blood vessels [48, 49]. Macrophages also modulate the bioavailability of VEGF-A in tumours through processing by matrix metalloproteinases [50]. In addition, antibody-mediated neutralisation of angiopoietin 2, the ligand for the Tie2 receptor, or macrophage depletion blocks tumour angiogenesis and limits tumour progression in a mouse model of breast cancer [51, 52].

## 4. Macrophages Promote Chemoresistance

**Figure 1:**
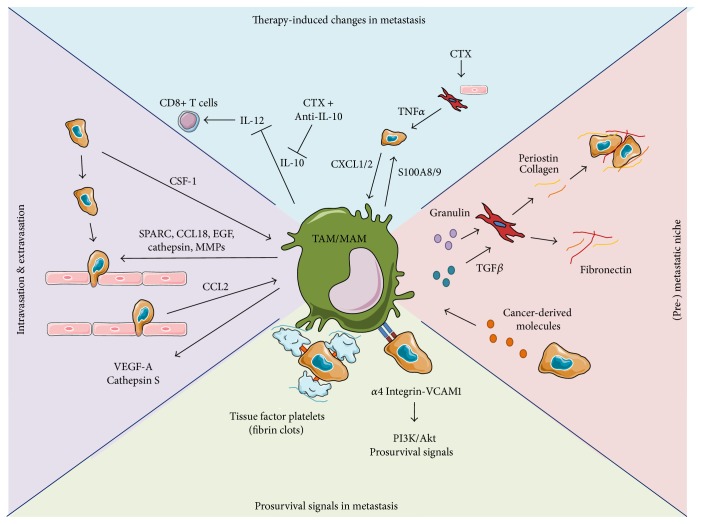
Prometastatic functions of macrophages. Macrophages promote invasion and intravasation of tumour cells at the primary site (*purple*). Tumour cells produce CSF1 that induces EGF expression in TAMs. This autocrine loop leads to comigration of tumour cells and macrophages towards blood vessels where macrophages produce VEGF-A to promote increased vessel permeability. In addition macrophage-derived molecules such as SPARC, CCL18, and proteases promote increased tumour cell invasion and migration. At the metastatic site, tumour cell-derived CCL2 recruits inflammatory monocytes to the metastatic site, where they differentiate into metastasis-associated macrophages that produce VEGF-A and cathepsin S to promote cancer cell extravasation. Macrophages promote survival at the metastatic site (*green*). Macrophages express integrin *α*4 that engages VCAM1 on tumour cells at the metastatic site, which increases tumour cell survival through PI3K/Akt signalling. In addition, macrophages bind to fibrin complexes on tumour cell-associated platelets, which increase tumour cell survival in the initial phase of metastatic colonisation. Macrophages promote metastatic niche formation (*pink*). Metastasis-associated macrophages produce granulin that activates HSTC to produce ECM molecules, such as collagen and periostin, which enhances the colony formation abilities of cancer cells in the metastatic niche of pancreatic cancer. In addition, tumour-derived exosomes can activate TGF*β* expression in Kupffer cells that activates HSTCs to produce fibronectin in the premetastatic liver. Macrophages promote therapeutic resistance (*blue*). Macrophages produce IL-10 that inhibits the effector functions of CD8+ T cells by blocking the effects of dendritic cell-derived IL-12. Inhibition of IL-10 with a blocking antibody in combination with chemotherapy improves the therapeutic response. Tumour cells express CXCL1/2 that induces S100A8/9 production in macrophages to improve tumour cell survival. Chemotherapy induces TNF*α* expression from cancer-associated fibroblasts and endothelial cells that reinforces the CXCL1/2-S100A8/9 axis and limits the efficacy of chemotherapy.

Macrophages play a key role in therapeutic resistance to chemotherapy [53]. Cytotoxic therapies can induce tumour cell expression of CSF-1, which results in an increased macrophage infiltration. Blockade of CSF-1 and CSF-1R in combination with chemotherapy improved survival and reduced the metastatic frequency in a breast cancer model and this response correlated with an increase in cytotoxic CD8+ T cells within the tumours [24]. Macrophages induce the expression of cytidine deaminase, the primary metabolizing enzyme of the chemotherapeutical agent gemcitabine, in pancreatic cancer cells. This results in an increased tumour cell survival in response to chemotherapeutic treatment of orthotopically implanted pancreatic tumours, which could be prevented by inhibition of CCR2+ inflammatory monocytes or depletion of macrophages [54]. TAMs also promote chemoresistance in pancreatic cancer through insulin-like growth factor (IGF) 1 and IGF2. Antibody-mediated neutralisation of IGF in combination with gemcitabine improves the response to chemotherapy, which results in reduced tumour size and increased cancer cell apoptosis in an orthotopic pancreatic cancer model [55]. Chemotherapeutic agents can also directly induce the expression of cathepsins in macrophages. In this study, macrophage-derived cathepsins were sufficient to protect tumour cells from cell death and blockage of cathepsins restored the sensitivity of cancer cells to several chemotherapeutic agents [56]. An indirect mechanism of how macrophages increase chemoresistance was described by Lisa Coussens and colleagues [57]. In this study, macrophages were identified as the main source of IL-10. IL-10 was found to inhibit the expression of IL12 in dendritic cell and subsequently reduced the activation of cytotoxic CD8+ T cells. Interestingly, antibody-mediated neutralisation of IL-10 in combination with chemotherapy increased the sensitivity to chemotherapeutic treatments [57] (Figure 1).

## 5. Macrophages Promote Different Aspects of Metastasis

The final step of cancer progression is the development of distant tumours in different organs from where the cancer initially developed. This process referred to as metastasis is extremely clinically relevant since the vast majority of cancer patients die with metastatic tumours.

Metastasis is a series of steps that the tumour cells must go through before they develop into clinically detectable metastatic tumour lesions. At the primary site, cancer cells must invade the surrounding tissue and intravasate into blood and/or lymphatic vessels. This allows the cancer cells to circulate in the body and spread to secondary sites. The organisation of the circulatory system that moves blood around the body and the structure of the capillary walls in each organ influence the pattern of cancer cell metastasis. The circulating tumour cells become arrested in the capillaries at the secondary site and must extravasate from the vessel to initiate the colonisation. This part of the process can be divided into many steps that take place on a timescale of several years. After extravasation, cancer cells must develop resistance from the immune system and host-tissue defences. This is made possible by settlement in supportive niches that enables them to survive as micrometastases that are not possible to detect with current technology. It is also thought that the supportive niche can enhance tumour-stem cell traits that endow the tumour cells with the ability to reinitiate their growth and develop into clinically detectable macrometastases. In some cases, therapeutic treatment can partially eliminate the macrometastatic lesions, but this usually leads to survival of drug-resistant tumour cells through niche-mediated survival mechanisms that eventually relapse as a drug-resistant metastatic lesion [58–61]. Macrophages can promote each step of the metastatic cascade, which we will discuss in more detail in the following sections.

### 5.1. Premetastatic Niche

Systemic effects from a primary tumour that occur before tumour cell dissemination can prepare future metastatic site(s) and increase the efficiency of disseminated tumour cells (DTCs) colonisation [62]. Primary tumours produce factors such as lysyl oxidase, PlGF, and exosomes that prepare the secondary site for the arrival of disseminated tumour cells in what is termed the premetastatic niche. These tumour-derived factors induce the accumulation and programming of CD11b+VEGFR1+ myeloid cells that cluster at the secondary site before the arrival of tumour cells and promote metastatic colonisation upon DTC arrival [63–68].

Most studies have focused on recruitment of myeloid cells to the premetastatic niches, but resident macrophage populations also play a role in formation of the premetastatic niche. Interestingly, preconditioning of tumour-free mice through administration of conditioned medium from B16 melanoma cells, with a distinct metastatic profile towards multiple organs, could change the metastatic pattern of injected Lewis lung carcinoma cells, that primarily metastasise to the lungs, to include organs such as testis, spleen, and kidney, which is similar to the metastatic pattern of B16 melanoma cells [63]. This was later demonstrated to depend on tumour-derived exosomes, which are small membrane vesicles (30–100 nm) that contain functional biomolecules (such as proteins, lipids, RNA, and DNA) that can be horizontally transferred to recipient cells. Injection of tumour-derived exosomes from cells with specific metastatic patterns resulted in a metastatic distribution of injected tumour cells that matched that of the cell that had produced the exosomes. Specifically, pretreating mice with exosomes from lung-tropic cancer cells followed by injection of bone-tropic cancer cells resulted in increased lung metastasis of the bone-tropic cells. The exosomes from lung-, liver-, or brain-tropic cancer cells had distinct integrin expression profiles that were required for successful uptake by cells in the premetastatic site (such as endothelial and epithelial cells of the lungs, Kupffer cells in the liver, and endothelial cells in the brain, resp.). Knockdown of the individual exosomal integrins could inhibit organ-specific metastasis. The same research group demonstrated, in a mouse model of pancreatic cancer metastasis, that cancer-derived exosomes are taken up by liver-resident Kupffer cells. The exosomes contain macrophage inhibitory factor that induces transforming growth factor *β* production from Kupffer cells, which activate resident hepatic stellate cells (HSTCs) into myofibroblasts that prepare the liver for metastatic DTCs by production of fibronectin to recruit monocytes and macrophages [69, 70] (Figure 1). However, the ability of other resident macrophage populations, such as lung alveolar macrophages, to initiate premetastatic niche formation in the lung is yet unexplored.

### 5.2. Primary Tumour Invasion and Metastatic Extravasation

Macrophages promote invasion and metastasis from the primary tumour site through their ability to engage cancer cells in an autocrine loop that promotes cancer cell migration. This autocrine signalling involves CSF-1 production from the cancer cells that engage the macrophages to produce epidermal growth factor, which ultimately leads to comigration of macrophages trailed by cancer cells towards tumour blood vessels where macrophage-derived VEGF-A promotes cancer cell intravasation into the blood vessels [71–73]. In addition, macrophage-derived cathepsins, SPARC, or CCL18 enhances the tumour cell adhesion to extracellular matrix proteins and promotes tumour cell migration [74–76].

Macrophages orchestrate metastatic development by distinct cellular interactions within metastatic sites. Intravital microscopy of DTCs in the lungs immediately after tail vein injection reveals that DTCs are lodged inside the lung capillaries and begin to shed microparticles with an average diameter of 5 *μ*m due to shear forces in the lungs. These microparticles are taken up by neutrophils, monocytes, and macrophages at the metastatic site in three distinct waves within the first 24 hours after DTC arrest. In addition, CD103+ dendritic cells also take up microparticles and migrate to the lymph nodes. Ablation of CCR2+ monocytes and macrophages reduces the metastatic burden in the lungs. This correlates with increased microparticle loading in CD103+ dendritic cells and increased presence of CD8+ T cells in the lungs. In contrast, depletion of CD103+ dendritic cells results in increased metastatic development [77].

Macrophages promote extravasation of arrested DTCs in capillary networks at the secondary site. DTCs produce CCL2 that recruits inflammatory monocytes from the blood to the metastatic site. Here, inflammatory monocytes secrete VEGF-A to promote DTC extravasation through increased vascular permeability [78, 79]. In addition, CCL2 induces the expression of CCL3 from metastasis-associated macrophages (MAMs) that promote the retention of MAMs at the metastatic site. This improves the direct contact between cancer cells and macrophages through VCAM1-*α*4 integrin mediated signalling and promotes cancer cell retention in the metastatic site [80].

Both macrophages and tumour cells produce cathepsin S. High expression of cathepsin S in primary tumour samples from breast cancer patients correlates with decreased brain metastasis-free survival. Mechanistically, cathepsin S mediates blood-brain barrier transmigration through proteolytic processing of the junctional adhesion molecule, JAM-B, and only the combined depletion of both MAM-derived and cancer cell-derived cathepsin S reduces the development of brain metastasis [81] (Figure 1).

### 5.3. Colonisation

Once DTCs have extravasated, they find themselves in an unfamiliar environment where crosstalk between DTCs and their microenvironment is essential for successful metastatic colonisation. This allows DTCs to escape immune-mediated destruction and initiates niche-dependent survival signalling. One of the key components of the metastatic niche is macrophages that promote metastatic colonisation through various mechanisms.

Tissue factor expressed on DTCs can recruit platelets and activate the coagulation cascade which leads to thrombin activation and fibrin deposition (clot formation). Macrophages are recruited to the clots on extravasated DTCs in the lung and promote cancer cell survival in a NK cell-independent mechanism [82]. This might be due to the direct interaction between DTCs and MAMs, as it was demonstrated that macrophages promote DTC survival by initiating cell-cell contact. Here, DTCs, which have high expression of VCAM-1, engage *α*4-integrins on MAMs to initiate prosurvival signalling within cancer cells through the PI3K/Akt signalling pathway [83]. We recently demonstrated a crucial prometastatic mechanism of MAMs in pancreatic cancer to orchestrate the establishment of a metastatic niche in the liver. Inflammatory monocyte-derived MAMs accumulate in the liver upon DTC arrival. Pharmacological depletion of MAMs with clodronate liposomes or blockade of inflammatory monocyte recruitment through PI3K*γ* depletion, which is important for monocyte trafficking to inflammatory sites [84], decreased the metastatic burden and correlated with a reduction in alpha smooth muscle actin-positive (*α*SMA+) myofibroblasts. Mechanistically, we found that MAMs secrete granulin to activate resident HSTCs into *α*SMA+ myofibroblasts. The activated myofibroblasts produce extracellular matrix molecules such as periostin that enhances colony formation abilities of pancreatic cancer cells. Depletion of granulin in the bone marrow compartment ablated the deposition of extracellular matrix and periostin in the metastatic lesions and resulted in reduced proliferation of metastatic cancer cells [85]. A myofibroblast activating function of granulin has been previously reported in a breast cancer model. Here tumour-instigating cells promoted the outgrowth of contralateral implanted indolent tumour cells, through recruitment of granulin-secreting myeloid cells to the indolent tumour site, which correlated with accumulation of *α*SMA+ myofibroblasts [86]. In respect of macrophages and pancreatic cancer metastasis, a recent report showed that long-term pharmacological depletion of macrophages in the genetic KPC mouse model of pancreatic cancer (Pdx1^cre^; Kras^G12D/+^; p53^R172H/+^) [87] markedly reduced metastasis [88] (Figure 1).

## 6. Targeting Macrophage Functions in the Tumour Microenvironment

Because of the important role of macrophages in tumour development they have emerged as a promising therapeutic target (Figure 1). Among the potential strategies to inhibit macrophage function in the tumour microenvironment are (1) blocking their recruitment or depletion from the tumour, (2) reeducation to an antitumorigenic phenotype, or (3) immunostimulatory reactivation.

Since CSF-1 is the most important cytokine for macrophage survival, several strategies have been developed to block ligand binding to CSF-1R. These include antibodies that block CSF-1 or CSF-1R, thus preventing receptor ligation. Treatment with these antibodies has decreased tumour burden in several preclinical animal models [28, 89] and human patients [90]. One emerging strategy to inhibit macrophages is reeducation to an antitumorigenic M1-like phenotype. Interestingly, treatment with two different anti-CSF-1R antibodies resulted in a macrophage reprogramming in mouse models of glioma and pancreatic cancer. In both cases, antibody treatment reversed macrophage polarization from an M2 to an M1 profile through downregulation of markers associated with the M2-like phenotype and upregulation of markers associated with a M1-like profile [28, 91]. Macrophages can be recruited to tumour sites by tumour-derived CCL2 that binds to CCR2. Disrupting this axis by targeting CCR2 or CCL2 has resulted in reduced mobilization of inflammatory monocytes from the bone marrow and peripheral blood to tumour sites, which correlated with increased survival and decreased tumour burden in mouse models of lung metastasis and pancreatic cancer [78, 92]. In addition, the chemotherapeutic agent Trabectedin was reported to specifically deplete monocytes and macrophages in several animal tumour models resulting in reduced tumour angiogenesis and reduced tumour growth [93]. Furthermore, low-dose radiotherapy has been shown to reprogram macrophages to a M1-like profile that promotes the normalization of tumour vasculature and efficient recruitment of cytotoxic T cells in both mouse models and human patients with pancreatic cancer [94].

Finally, activation of macrophages in a genetically engineered mouse model of pancreatic cancer with an agonist monoclonal CD40 antibody synergizes with chemotherapy (CTX) to induce tumour regression [95]. These results were based on the ability of the CD40 antibody to enhance antigen presentation, deplete the desmoplastic stroma, and ultimately promote antitumouricidal activities of monocytes, macrophages, and CD8+ T cells before their recruitment to the tumour site [96, 97].

## 7. Targeting Macrophages at Metastatic Sites

Despite advances in cancer treatment, surgical removal of a tumour is still considered the best treatment if possible. Surgery is often complemented with systemic chemotherapy treatment before (neoadjuvant) or after (adjuvant) surgical resection. Neoadjuvant chemotherapy aims to reduce tumour burden, thereby allowing surgical intervention, whereas adjuvant chemotherapy is standard of care treatment and aims to eliminate residual cancer cells at the surgical site or clinically undetectable metastatic deposits. However, this treatment may fail due to niche-mediated survival at either site [60]. From that point of view it might be beneficial to target the prometastatic stromal compartment, including macrophages in combination with current cytotoxic regimens, which mainly target cancer cells. Indeed, several inhibitors of the CSF1-CSF-1R or CCR2-CCL2 signalling axes have shown therapeutic benefits in mouse models of pancreatic and breast cancer, both in combination and without chemotherapeutical agents [28, 78] and in clinical settings [90, 92, 98]. However, further work must determine the optimal treatment conditions, since cessation of treatment may have detrimental effects as recently demonstrated for anti-CCL2 antibodies. Experimental neutralisation of CCL2 with anti-CCL2 antibodies in mouse models of breast cancer metastasis, although limiting early metastatic processes, promoted metastasis following the cessation of therapy. Ending treatment increased the mobilization of inflammatory monocytes and their recruitment to micrometastatic deposits, which increased angiogenesis and metastatic proliferation through VEGF-A and IL-6 [99]. For patients with inoperable disease, systemic treatment is the only available treatment, but efficiency is limited by development of drug resistance [60]. In a breast cancer model, CXCL1/2 is produced by cancer cells and serves as a chemoattractant for myeloid cells that are recruited to the lungs, where they produce S100A8/9 to enhance cancer cell survival at the metastatic site. Treatment of mice with the chemotherapeutic agents Doxorubicin and Cyclophosphamide enhanced the CXCL1-S100A8/9 axis. Interestingly, this amplification was due to the direct effect of chemotherapy on endothelial cells and fibroblasts that produced TNF*α* to stimulate further CXCL1/2 production from the cancer cells [100]. Treatment with anti-CSF-1R antibodies reprograms macrophages in a glioma mouse model to a M1-phenotype and limits tumour growth. However, macrophages in the tumour microenvironment became refractory to the effect of anti-CSF1R antibodies resulting in regrowth of glioma tumours. This was caused by IGF1 production from macrophages stimulated with CD8+ T cell-derived IL-4 [91, 101]. Furthermore, treatment with neutralising anti-CSF-1R or anti-CSF1 antibodies can lead to a compensatory increase in granulocyte colony stimulating factor (CSF3), which stimulates an increase in neutrophils at the primary tumour site and in metastatic deposits. The increased neutrophil accumulation results in increased metastatic development, which could be prevented by the addition of a neutralising anti-CSF3 antibody in combination with the anti-CSF1 antibody [102].

It was believed that directing the tumour microenvironment might serve as a more promising therapeutic target than the cancer cells compartment due to decreased likelihood of developing therapeutic resistance through mutations in the targeted cells with the tumour microenvironment. These reports stress the need for more research into the role of cells in the tumour microenvironment, especially the macrophages, both in response to targeted therapies and without.

## 8. Future Directions

Macrophages are essential components of all mammalian tissues where they perform a variety of supportive functions that reaches beyond their classical functions as antimicrobial phagocytes. However, the molecular mechanism of how the origin of macrophages and their tissue specificity affect their tumour promoting and/or tumour suppressive functions still remains poorly understood. Macrophages have high plasticity and their biological functions can differ markedly based on their organ/tissue specificity. Transcriptional factors have been identified that control the differentiation of progenitor cells into macrophage, while different transcriptional factors can be induced in an organ-specific manner, thereby regulating macrophage identity relative to their ascribed function within that organ. It will be interesting to further characterise the relative contribution of transcriptional programs induced by tissue-derived signals versus signals regulated by a functional demand in the tumour microenvironment (such as hypoxia or tumour-derived signals). This might be particularly important for certain cancer types and could possibly reveal new tumour-promoting mechanisms and offer new therapeutic targets to inhibit protumourigenic macrophages.

We and others have described different mechanisms of how metastasis-associated macrophages promote metastasis by mediating cancer cell extravasation, stimulating cancer cell survival signalling pathways, and inducing metastatic niche formation at the secondary site after tumour cell dissemination. Tissue-resident macrophage populations seem to play a role in the initial phase of the premetastatic niche formation, but their role in metastatic progression at the secondary site remains unexplored.

While macrophages remain a promising therapeutic target in multiple cancer types, recent reports concerning acquired resistance in different tumours to therapeutic agents that specifically target macrophages, such as anti-CCL2/CCR2 or anti-M-CSFR, highlight that it will be important to characterise potential resistance mechanisms when we develop agents that target macrophages in the tumour microenvironment.

## Figures and Tables

**Table 1:** Markers used to identify murine macrophage populations in healthy and tumour-bearing mice.

Type	Population	Markers	Reference
Healthy tissues	*Microglia* (During embryonic development)	CD11b^+^ CX3C1^+^ F4/80^+^ CSF1R^+^ Gr1^−^ F4/80^+^	Ginhoux et al., Science [11]
	*Alveolar macrophages* (Adult)	CD11b^+^ F4/80^+^SiglecF^high^CD11c^high^ CD64^+^	Guilliams et al., JEM [12]
	*Colon macrophages*		
	Embryonic	CD45^+^ Siglec-F^−^ Ly6G^−^CD11c^low^ CD64+ CD11b^low^F4/80^high^	Bain et al., Nature Immunology [15]
	Adult	CD45^+^ Siglec-F^−^ Ly6G^−^CD11c^low^ CD64+ CD11b^+^F4/80^low^	

Blood-derived cells in adult mice	*Patrolling monocytes*	Ly6C^low^CX3CR1^high^ CCR2^−^ CD62L^−^CD43^high^	Auffray et al., Science [19]
	*Inflammatory monocytes*	Ly6C^high^CX3CR1^mid^ CCR2^+^ CD62L^+^CD43^low^	Auffray et al., Science [19]
	*Neutrophils*	CD45^+^ CD11b^+^ F4/80^−^ Ly6C^+^Ly6G^high^	DeNardo et al., Cancer Disc. [24]

Tumour associated macrophages (TAM)	*Breast cancer* (MMTV-PyMT Model)		
	TAMs	CD11b^low^MHCII^high^ CCR2^+^ F4/80^+^ CD64^+^ MerTK^+^	Franklin et al., Science [31]
	Mammary-resident macrophages	CD11b^high^MHCII^high^	
	*Breast cancer* (MMTV-PyMT Model)		
	TAMs	CD11b^+^ Gr1^−^ F4/80^+^	DeNardo et al., Cancer Cell [37]
		CD45^+^ CD11b^+^ Ly6G^−^Ly6C^low^ F4/80^+^	DeNardo et al., Cancer Disc. [24]
		CD11b^+^ F4/80^+^ MHCII^+^ Ly6C^−^	Ruffell et al., Cancer Cell [57]
	*Breast cancer*		
	Subcutaneous N202 mammary tumors		
	Tie2-expressing monocytes (TEM)	CD45^+^ CD11b^+^ F4/80^+^ Tie2^+^ CD31^−^	DePalma et al., Cancer Cell [103]
	TAMs	CD45^+^ CD11b^+^ F4/80^+^ Tie2^−^ CD31^−^	Pucci et al., Blood [104]
	*Pancreatic cancer*		
	TAMs (Orthotopic KPC)	CD11b+ F4/80+ Gr1- MHCII^+^CD206^high^	Zhu et al., Cancer Research [28]
	TAMs (KC model; p48-CRE/LSL-KRas^G12D^)	CD11b+ Gr1-	Clark et al., Cancer Research [105]
	*Glioma* (PDGF-B–driven glioma)		
	TAMs	CD45^+^ CD11b^+^ CD68^+^ CSF1R^+^ Gr1^−^	Pyonteck et al., Nat Med [91]
